# How People Integrate Mental Health Apps Into Everyday Self-Care: Qualitative Study

**DOI:** 10.2196/95414

**Published:** 2026-10-09

**Authors:** Vít Suchý, Zuzana Scott, Barbora Boštíková, Marek Janoušek, Eliška Josková, Anna Ševčíková

**Affiliations:** 1 Psychology Research Institute, Faculty of Social Studies, Masaryk University, Jostova 10, Brno, 60200, Czechia, 420 549 49 4393; 2 Department of Psychology, Faculty of Social Studies, Masaryk University, Brno, Czechia

**Keywords:** mental health app, self-care, mental health, adults, qualitative study, mobile applications, self-management, patient engagement, habit

## Abstract

**Background:**

Mobile mental health (MH) apps are increasingly promoted as tools for self-care. However, less is known about how users integrate them into everyday life and how they combine with other forms of support.

**Objective:**

This qualitative study examined what motivates individuals to adopt MH apps and how these tools are used alongside other care practices.

**Methods:**

We conducted semistructured interviews with 31 Czech MH app users aged 18‐56 (23 women), recruited through social media, researchers’ networks, and snowball sampling. Eligible participants had used an MH app for at least 2 weeks; individuals currently experiencing a psychological crisis were excluded. Interviews were conducted online or face-to-face and analyzed using reflexive thematic analysis by Braun and Clarke. The analysis was inductive and informed by a critical realist epistemological stance. Initial analytic engagement was conducted independently, followed by collaborative theme development and refinement.

**Results:**

We identified the following three motivational patterns for MH app use: (1) regular use to maintain well-being and support preventive self-care; (2) reactive use during periods of acute distress; and (3) temporary, problem-focused use aimed at understanding or addressing a specific difficulty. Beyond motivations for adoption, our findings revealed the following four distinct trajectories of app engagement and disengagement: (1) emancipation, with apps acting as ongoing support for self-care when therapy is reduced or ended; (2) independence, as techniques learned through apps are transferred into everyday life and used without the app; (3) reliance, where users persist with app-based self-care despite insufficient benefit, occasionally delaying or limiting other forms of help; and (4) episodic use, characterized by intermittent engagement during specific life periods. These findings extend existing research by showing that disengagement is not necessarily a marker of failure but can reflect successful self-care and the transfer of learned skills. We also identified tensions and unintended consequences, including the burden of sustaining engagement, risks of overreliance, emotional costs associated with sustained self-monitoring, and tensions between digital self-care and efforts to reduce screen use. Participants generally viewed self-monitoring as beneficial when guided by clear personal goals and embedded within a broader system of self-care.

**Conclusions:**

Our findings suggest that appropriate disengagement can signal a successful step in self-care. App design and policy should acknowledge episodic use and strengthen connections to human support with clear guidance and accessible pathways to additional care when needed.

## Introduction

### Background

A growing number of studies point to a rising prevalence of mental health (MH) problems in the adult population [1-3]. In parallel, broader social processes of individualization in Western countries increasingly frame MH and well-being as an individual responsibility [4]. This orientation is reflected in the individualization of care, whereby individuals are expected to monitor their well-being and manage psychological difficulties largely on their own [5]. Recent technological advances have further reinforced this process through the rapid development of stand-alone MH smartphone apps designed to improve users’ well-being.

These trends matter most where formal care is hard to access. In Czechia, roughly 80% of adults meeting criteria for a current mental disorder receive no treatment, and over half of those seeking help encounter structural barriers [6]. Public stigma also remains high by European standards, with little recent change even as recognition of conditions like depression and anxiety has modestly improved [7]. In such contexts, MH apps may be adopted out of necessity rather than personal preference.

An expanding body of systematic reviews suggests that these digital tools may have moderate positive effects on reducing negative MH symptoms, improving well-being and emotional regulation [8-13], particularly with regard to depression and anxiety-related symptomatology. Despite this emerging evidence, numerous studies highlight persistent challenges related to low user engagement and factors that hinder the sustained use of MH apps [9,14,15]. Recent research therefore suggests that greater attention should be paid to personalization mechanisms within MH apps as promising strategies to enhance use of and perceived benefits from these digital tools [15,16]. However, significant gaps remain in our understanding of when and how apps benefit users and what role they play in broader self-care practices.

### Users’ Motivations and Experiences of MH App Use

Prior qualitative research has identified several factors motivating individuals to use MH apps. App properties that enable users to substitute emotional support usually provided by social interactions have often been highlighted. Chief among these is their immediate availability, which allows users to address psychological distress independent of the availability of social support. MH apps also provide a discreet space where users can address their MH needs. This privacy is particularly relevant in cases where stigma discourages the disclosure necessary for social support [17-19]. In addition to privacy, MH apps are often valued for their nonjudgmental stance. This provides some users with a sense of being understood and supported, mitigating feelings of loneliness and/or shame, while they remain aware of the app’s nonhuman nature [17-19].

Beyond these substitutional properties, users are attracted by the diverse features (eg, breathing exercises and journaling) consolidated within single customizable platforms, which are often economically affordable [17,19-21]. This is an important factor for sustained app usage, particularly because MH apps have been found to be valuable for long-term MH maintenance and for allowing more precise and continuous tracking for mood and daily circumstances, thereby fostering self-awareness of the factors associated with changes in psychosocial functioning [15,18,22]

However, existing research indicates that engagement features, such as streaks and reminders, are often experienced ambivalently; while they may encourage routine for some, the perceived burden of continuous tasks can paradoxically discourage use [15,22]. Furthermore, concerns raised by MH app users relate to potential overreliance on app-based functions, particularly in the absence of complementary forms of support [19,23]. According to some users, this reliance contributes to the emergence of a new norm of independent coping, and failing to conform to this expectation leaves them with feelings of shame [24].

Current research also suggests that sustained, long-term use of MH apps for self-maintenance and health management requires a high level of self-initiative and intrinsic motivation [15]. These demands become compounded when users experience psychological distress, which may make it more difficult for them to familiarize themselves with app content or to effectively navigate available features [15]. In this regard, the involvement of external professional support from counselors and therapists to assist users in engaging with and interpreting MH app content has been identified as a potentially promising approach [19].

A recent qualitative study shifted the research focus from the sustained use of MH apps to macro-engagement, within which off-screen, goal-directed activities associated with behavioral change may continue beyond app use [25]. This study showed that on-screen interactions and off-screen, goal-directed practices often remained disconnected, which limited the extent to which behavioral change was supported in everyday life. The authors therefore argued that greater support for engagement beyond the screen was needed. However, aside from this emerging line of research, we know little about how people integrate MH apps into their broader MH care practices and how these digital tools interact with the other forms of support with which people engage.

### Theoretical Framework for Self-Care

To examine the use of MH apps, we draw on the Middle-Range Theory of Self-Care of Chronic Illness (MRTSC) proposed by Riegel et al [26]. Within this framework, self-care is conceptualized as an ongoing process aimed at maintaining health and well-being in both healthy and ill states. The theory distinguishes the following three interrelated aspects: (1) *self-care maintenance*, which involves behaviors intended to preserve health and prevent symptom exacerbation; (2) *self-care monitoring*, which refers to the observation and tracking of bodily or psychological changes; and (3) *self-care management*, which encompasses responses to symptoms based on their detection and interpretation. This characterizes self-care as a decision-making process during which individuals reflect on their symptoms based on experience and contextual factors.

Although MH self-care differs from self-care in chronic illness in that needs and goals may fluctuate over time, the MRTSC framework provides a useful lens for understanding how individuals maintain well-being, monitor psychological states, and respond to perceived changes in their MH. The framework is particularly relevant for studying MH app use, as these apps can facilitate all 3 aspects of the self-care process. They support monitoring and reflection through features such as mood tracking or journaling; they enable self-care maintenance through guided relaxation or mindfulness practices; and they assist in self-care management through actionable coping strategies or crisis resources. Moreover, the MRTSC offers a process-oriented framework that is particularly suited to symptom-driven engagement and decision-making in MH self-care contexts. This perspective allows MH app use to be understood as part of an ongoing self-care process alongside other forms of professional and informal support. In this respect, understanding the use of MH apps as part of self-care raises important questions about when, how, and for how long individuals integrate these tools into their everyday practices.

### This Study

Existing research on MH apps remains largely descriptive and fragmented—motivations, user engagement, and disengagement are often examined in isolation. Moreover, disengagement is often interpreted as a negative outcome. Recent human-computer interaction research [27] offers an alternative perspective by conceptualizing disengagement as a natural cyclical phase of technology use that is driven by user agency and specific goals. Pausing or stopping use can be a deliberate positive decision rather than a breakdown in motivation or usability. Applying this framework may help explain how disengaging from MH apps may reflect successful self-care management.

Furthermore, while recent research has begun to highlight the disconnect between on-screen engagement and off-screen behavioral change [28], less attention has been paid to how MH apps are embedded within broader ecosystems of MH care and support. To address this gap, we draw on the MRTSC to examine how users engage with and disengage from MH apps and how these apps are embedded within broader MH self-care practices and support systems. To achieve this aim, the study addresses the following research questions (RQs):

- RQ1: What motivates individuals to engage with and disengage from the MH apps?
- RQ2: How are MH apps integrated into users’ everyday lives and into broader ecosystems of MH care and support?

## Methods

### Participants and Procedure

We conducted 31 semistructured interviews with Czech users of mobile MH apps (aged 18‐56, 23 women). Recruitment was initially conducted through a paid social media advertising campaign (Facebook [Meta] and Instagram [Meta]) targeting the general Czech-speaking population in Czechia aged 18 years and older. Owing to low response rates to the advertisement, recruitment was subsequently supplemented through the researchers’ personal networks and further through snowball sampling. None of the participants had a prior relationship with the interviewers. We were transparent about the study’s purpose, framing the conversation as an exploration of how people use these apps, their benefits, and the boundaries of their usefulness. We excluded individuals who were (1) 17 years old or younger, (2) currently in psychological crisis, or (3) survivors of a traumatic event within the preceding 12 months. The only inclusion criterion was having used a MH app for at least 2 weeks. Demographic data were collected before the interview (Table 1).

**Table 1.** Sample characteristics (n=31).

Characteristic	Value
Age (y), mean (SD)	28.81 (9.58)
Interview length (hours:minutes:seconds), mean (SD)	0:57:45 (0:17:05)
Gender, n (%)	
Women	23 (74.2)
Men	8 (25.8)
Nationality, n (%)	
Czech	30 (96.8)
Slovak	1 (3.2)
Size of municipality, n (%)	
>500,000	8 (25.8)
100,000‐500,000	11 (35.5)
20,000‐100,000	3 (9.7)
10,000‐20,000	2 (6.5)
<10,000	7 (22.6)
Education, n (%)	
Primary	2 (6.5)
Secondary	7 (22.6)
Tertiary	22 (71)
Marital status, n (%)	
Single	20 (64.5)
Divorced	2 (6.5)
Widowed	0 (0)
Married	9 (29)
Relationship status, n (%)	
Single	13 (41.9)
In a relationship	18 (58.1)
Number of children, n (%)	
0	30 (96.8)
2	1 (3.2)
Application used, n (%)	
Nepanikař	11 (22.4)
VOS	7 (14.3)
Calm	4 (8.2)
Finch Care	4 (8.2)
Headspace	3 (6.1)
Waking up	2 (4.1)
Calmio	2 (4.1)
Apple Health	2 (4.1)
How We Feel	2 (4.1)
Other	12 (24.5)
Recruited through, n (%)	
Recruitment advert	15 (48.4)
Snowball or personal contact	16 (51.6)
Difficulty in making monthly ends meet (last 12 mo), n (%)	
Each time with difficulty	2 (6.5)
Usually with difficulty	1 (3.2)
Occasionally with difficulty	7 (22.6)
Rarely with difficulty	10 (32.3)
Completely without difficulty	11 (35.5)
Interview format, n (%)	
Face-to-face	11 (35.5)
Online	20 (64.5)

The semistructured interview guide was developed through a multistage, iterative process. Following agreement on the research objectives and questions, all the authors of this study independently proposed thematic domains and interview questions relevant to the investigated phenomenon. Through discussion and consensus, these suggestions were consolidated into 11 thematic areas such as participants’ app use, motivations, perceived benefits and drawbacks, comparisons with other forms of MH support, trust, and long-term experiences with MH apps. The preliminary guide was further informed by a screening of MH apps available in major app stores and an analysis of user reviews, which helped identify salient user experiences and recurring concerns. Finally, the guide was pilot tested with three individuals (1 woman aged 26 y and 2 men aged 25 and 27 y) who had experience using MH apps. Based on participant feedback, several questions were reworded, alternative prompts were added, and items perceived as overly broad or insufficiently relevant to participants’ experiences were refined. The full interview guide, including translations, is available in Multimedia Appendix 1.

### Ethical Considerations

The study received ethical approval from the Masaryk University Research Ethics Committee (0198/2025) prior to data collection. All participants provided written informed consent and consent for the processing of personal data. Informed consent was obtained from all participants in writing and reaffirmed verbally immediately before the interview. Participation was voluntary, and participants could withdraw from the study without penalty before their data were anonymized. Interviews were audio-recorded and transcribed for analysis. Audio recordings were stored securely and deleted following transcription. Transcripts were anonymized prior to analysis, and access to study data was restricted to authorized members of the research team. Participants received CZK 500 (equivalent to US $23.23) as compensation for completing the interview. One participant described past suicidal ideation but indicated during the interview that these thoughts were no longer current. At the end of the interview, the interviewer returned to this disclosure, checked whether the participant wanted information about relevant support services, and offered to provide contact information.

### Data Analysis

We conducted reflexive thematic analysis (RTA), following the 6-phase approach outlined by Braun and Clarke [29]. The MRTSC informed the broader conceptual framing of the study and was used as a lens through which the findings were subsequently interpreted. RTA is a flexible method suited to exploring patterned meaning across participant accounts. Our epistemological stance was informed by critical realism [30], which assumes that the participants’ accounts provide access to real experiences and underlying mechanisms, while recognizing that these experiences are always mediated through language, context, and interpretation. The analysis was inductive and bottom-up, with themes developed from the data rather than imposed a priori. Consistent with RTA, data saturation was not used as a stopping criterion; instead, recruitment concluded after the project’s predefined recruitment target had been reached and the dataset was judged sufficiently rich and varied to address the RQs [31]. Participants provided detailed accounts of their experiences, and the sample encompassed diverse patterns of app use, motivations, perceived benefits, challenges, and trust-related concerns, enabling the development of well-supported themes addressing the study aims. The adequacy of the dataset was assessed throughout the iterative process of data collection and analysis. Toward the end of recruitment, additional interviews continued to provide illustrative examples and nuances of participants’ experiences but did not substantially expand the range of issues relevant to the RQs. Given the breadth, depth, and diversity of the dataset, the research team concluded that it was sufficiently rich to address the RQs. Only authors VS, ZS, and AŠ were involved in the analytic process; stages 1‐3 (ie, data familiarization, initial coding, and early pattern identification) were conducted independently by each author to encourage diverse readings of the data, with regular consultation to discuss developing interpretations, while later stages of theme development, refinement, and definition were collaborative. This iterative and reflexive process allowed us to move between participant accounts and broader explanatory interpretations, attending both to surface meanings and to the underlying conditions that shaped app use and the consequences of app use. Further methodological specifics are reported in the accompanying COREQ checklist (Checklist 1).

### Positionality

AŠ and VS approached the analysis from backgrounds in critical health psychology, with particular attention to the interplay between individual self-care and broader health and care contexts. AŠ had limited personal experience with MH apps, while VS had previously used meditation apps before transitioning to unguided practice. ZS approached the research from a critical psychology perspective, with particular sensitivity to the social contexts in which individual responsibility for MH and well-being is constructed. These differing positions informed reflexive discussions of developing interpretations throughout the analytic process. A more detailed description of each author’s positionality is available in Multimedia Appendix 2.

## Results

### Sample Description

Regarding the duration of use, most of the sample had been using their apps of choice for over a year (n=21), followed by participants who had used them for 6 months to 1 year (n=6), and 2-6 months (n=4). Out of 31 participants, 10 disclosed MH issues that could be classified as symptoms related to a clinical disorder. Specifically, 5 participants reported panic attacks, 4 reported depressive mood, 3 reported anxiety, and 1 reported suicidal ideation. Clinical diagnoses disclosed during the interviews were attention-deficit/hyperactivity disorder (ADHD; n=2), bipolar disorder (n=1), and a history of alcoholism (n=1). For further sample characteristics, refer to Table 1.

### Analytical Results

Our analysis generated 3 themes that capture the dynamic nature of MH app use. These themes illustrate how participants initially engaged with apps, how use was reshaped or interrupted over time, and how MH apps were integrated within broader self-care strategies. The themes are analytically distinct but closely intertwined, reflecting self-care decision-making processes (Figure 1).

**Figure 1.**
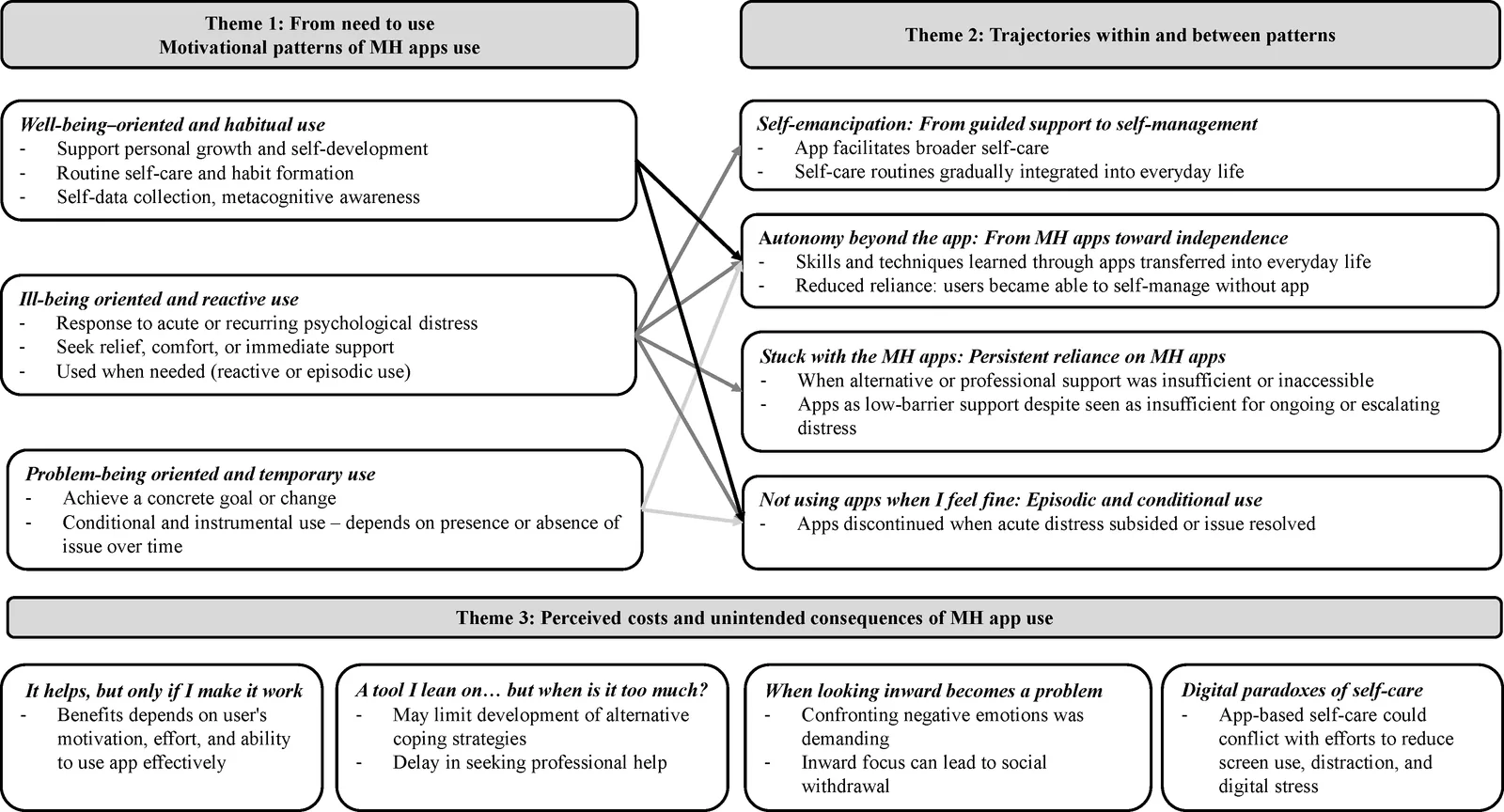
Motivational patterns, trajectories, and perceived costs of mental health (MH) app use. The figure provides a visual representation of the thematic findings. Arrows illustrate relationships between patterns (theme 1) and trajectories of MH app use (theme 2) identified in participants’ accounts. Theme 3 represents perceived costs and unintended consequences that cut across patterns and trajectories.

### Theme 1: From Need to Use—Motivational Patterns of MH App Use

#### Overview

Throughout the interviews, participants described diverse motivations for downloading MH apps. We identified 3 overarching motivational patterns that reflected how participants understood their mental well-being and the role of digital self-care at the onset of MH app use*—well-being–oriented and habitual use*, *ill-being-oriented and reactive use,* and *problem-oriented and temporary use*.

#### Well-Being–Oriented and Habitual Use

Well-being–oriented use was typically described as proactive and oriented toward maintaining or enhancing psychological well-being over time. Participants who entered app use from this position often framed MH apps as tools for routine self-care, supporting habit formation and reflection, and structuring time devoted to oneself. Most commonly, it was associated with functions that enabled self-data collection (eg, emotion tracking and journaling) or supported the development of metacognitive awareness (through meditation) and general mindfulness.

For several participants, well-being–oriented use also involved tracking functions to facilitate long-term reflection on life events and experiences. In these cases, participants wanted to document and preserve (mostly positive) personal life experiences over time:

*...to analyze my states or situations I find myself in... so I’ll be able to look back in history and analyze it, even learn from it, from mistakes or from what’s repeating.*

[P10_10_56M]

Importantly, well-being–oriented engagement did not always originate from a clearly articulated personal need. In some cases, app use began through contextual exposure, such as social media recommendations, employer-provided access, or professional curiosity among health and social care workers who sought to familiarize themselves with MH apps to assess whether to recommend them and which apps to recommend to their clients. What initially started as experimentation evolved into a meaningful self-care practice if participants experienced the app as beneficial.

The perceived value of habitual use often became particularly salient when use was interrupted. Some participants reported that during unintended breaks, most commonly due to lack of time, they felt less emotionally regulated or more scattered:

*I miss it in the sense that I know I approach life differently and I’m less emotionally stable.*

[P32 _27M]

These experiences functioned as a feedback loop that reinforced motivation to resume regular self-care practices, with continued support from MH apps.

#### Ill-Being–Oriented and Reactive Use

By contrast, ill-being–oriented use was characterized by reactive engagement with apps in response to acute or recurrent psychological distress. Participants turned to apps because of anxiety, panic attacks, emotional breakdowns, mood deterioration, or periods of intense stress. In these situations, apps were valued primarily for providing rapid relief in the form of distraction (eg, breathing techniques and simple interactive tasks) or grounding when other forms of support were unavailable. One participant compared having an MH app on her phone to:

*... [having] a pain killer in your medicine cabinet. One doesn’t need it every day, but you know if your head hurts really bad, it’s there.*

[P29_23F]

A recurring context for reactive use involved situations in which participants felt trapped or unable to leave, with MH apps described as a “lifebuoy” during periods of severe panic because they interrupted escalating anxiety and validated emotional experiences:

*...when the panic attack started to come on, I wasn’t able to think rationally... And I really thought I was going to suffocate. And I knew there were some breathing exercises there, and different exercises to kind of redirect attention, or the app tries to stop the brain from thinking ‘I’m dying, help, help...?*’

[P29_23F]

Ill-being–oriented use was typically episodic, with participants reporting that they stopped using the app once the acute distress subsided. As one interviewee noted:

*...the last time [I used the app] was about two weeks ago, so in terms of frequency now... maybe about once every two weeks. Honestly, I’m actually feeling quite well right now. Psychologically, I mean.*

[P14_19F]

Although apps were often described as effective in reducing immediate distress, participants generally distinguished this form of use from longer-term psychological work. Apps were framed as first aid or temporary support, rather than substitutes for therapy or deeper interventions.

#### Problem-Oriented and Temporary Use

The third motivational pattern reflected a problem-oriented approach to MH app use. Participants did not turn to apps during acute episodes but when they sensed that “*something was not right*” and wanted to understand or manage a specific issue over time. Their use of MH apps was framed less as a tool for immediate emotional regulation and more as a resource for understanding the issue. For example, one participant described using a different app for mood tracking to evaluate the emotional impact of her work, explicitly framing the app as a way to *see* and confirm or deny what she had been sensing:

*I was using [the app] because of work, because I wanted to find out whether that job was really affecting me emotionally so much, and how bad it actually was. ...I wanted to have it on paper and see to what extent it was the job and to what extent it was something else.*

[P12_34F]

Engagement with the apps was typically conditional and instrumental. It fluctuated over time, based on the presence or absence of the issue. Participants described adjusting the frequency and intensity of app use in response to changing circumstances or perceived need. Rather than striving for daily use, they deliberately returned to the app during more demanding periods and disengaged when conditions improved. As one participant noted, regular use felt unnecessary outside of more difficult phases:

*Now I only come back to [the app] when I feel like I need it. When there’s a more difficult period, I feel that it’s needed more to become more aware again.*

[P18_31F]

These motivational patterns captured the distinct ways in which participants entered MH app use. They differed in urgency, temporal framing, and expectations for what the apps could provide. However, our data also showed some overlap between these patterns, and some accounts revealed movement between patterns over time. Rather than discrete categories, the patterns are best understood as orienting distinctions that shaped how participants integrated the apps into their lives and the roles the apps played. How these motivational patterns evolved was the focus of the trajectories described further in this study.

### Theme 2: Trajectories Within and Between Patterns

#### Overview

Across interviews, participants narrated common variations in how they put the apps to use, reflecting shifting needs and life circumstances. We identified four trajectories to describe how participants moved within and between motivational patterns as follows: (1) the *self-emancipatory trajectory*, in which app use supported a shift from distress toward self-management; (2) the *trajectory from MH apps toward independence*, in which managing issues without the app was framed as a positive outcome; (3) the *persistent reliance trajectory*, characterized by continued or long-term use; and (4) the *episodic and conditional trajectory*, in which apps were used selectively, often only during periods of difficulty.

#### Self-Emancipation: From Guided Support to Self-Management

The first trajectory illustrates how MH app use could support a shift from distress management toward broader self-care routines. Here, autonomy was narrated not primarily as disengagement from the app, but as a gradual movement away from external support and toward more self-directed self-management. Specifically, some participants described transitions from ill-being- to well-being–oriented use, in which MH apps functioned as tools to facilitate the shift. Several participants described installing apps when they decided to reduce or terminate psychotherapy after reaching their initial therapeutic goals:

*...I think I kind of felt that I didn’t need [therapy] that much anymore, so maybe I perceived [the app] as some kind of replacement for therapy, that I still had some kind of certainty that, if something happened, it could maybe help me.*

[P06_23F]

This excerpt illustrates how MH apps could function as a psychological safety net, facilitating the transition from psychotherapy to more autonomous self-management.

In this regard, MH apps were often perceived as tools that created a visually appealing and user-friendly space. They legitimized the time devoted to self-care practices and thereby supported the development of self-care management. One interviewee further described how self-care subsequently extended beyond therapy:

*I’d say that actually [I do] a huge number of things that maybe aren’t a priori, that one would normally say are good for mental health[...] So, when a person reflects on it once in a while, or even every day, it at least then leads me to the point where, at a minimum, I’m reminded every day that I should be doing something for myself.*

[P18 _31F]

In this trajectory, MH apps supported the development of self-care routines that were subsequently integrated into participants’ everyday lives. Participants described a broader reorientation toward caring for their MH on a day-to-day basis, with apps serving as scaffolding during periods of transition.

#### Autonomy Beyond the App: From MH Apps Toward Independence

Across both ill-being and well-being pathways, some interviewees described a gradual reduction in their reliance on MH apps as they learned and internalized skills and techniques introduced through app use. They subsequently applied them independently in everyday life:

*[...] so it happens that I just tell myself,* “*Okay, I’m going to meditate for a few minutes even without the app.”*

[P16_52M]

For some interviewees, this transfer of skills into everyday life emerged as a positive side effect of MH app use, whereas others, such as the above-mentioned participant, described it as an explicit goal to become more mindful in daily life and to integrate techniques learned through the app beyond the app context.

Trajectories toward independence were characterized by an orientation toward transferring app-introduced skills into everyday life. For well-being–oriented users, this trajectory was motivated by a desire to integrate app-based practices more broadly into existing self-care routines. For those entering through an ill-being–oriented pathway, independence reflected an effort to avoid exclusive reliance on the app and to feel capable of managing situations without app-based guidance. For problem-oriented users, independence meant gaining sufficient understanding of a specific issue, allowing them to scale back or discontinue monitoring.

#### Stuck With MH Apps: Persistent Reliance on MH Apps

Not all trajectories led toward increased autonomy. For some users, the transition from MH app use as an emergency lifeline to the development of broader self-care practices did not materialize. These participants either maintained reliance on MH app techniques that had previously alleviated acute stress or continued to use MH apps as de facto substitutes for crisis helplines without further developing self-care practices. In both situations, they remained oriented toward MH apps not because the benefits were increasing, but because alternative forms of support were insufficient or perceived as inaccessible.

For 1 participant, escalating stressors eventually rendered app-based coping insufficient:

*But even then, when I walk outside [after MH app use], the first thought just goes back to the original problem and I’m right where I was before, so basically it doesn’t really work anymore. I don’t want to bash the app. It’s more that I’m currently experiencing much more stress, and in a more long-term way, and it’s not just one isolated problem.*

[P17_38M]

In this interview, seeking help from health care professionals was not considered, despite the perceived insufficiency of app-based support.

In cases where apps substituted for crisis intervention, especially free versions with limited functionalities, they functioned as low-barrier support for individuals experiencing psychological distress and limited financial means while simultaneously attempting to access professional care:

*[...] either calling the crisis hotline, which has happened to me several times when I couldn’t get through... I definitely looked for [the app] myself, because I had long known that I needed psychotherapy. I couldn’t afford that, so I tried to find free psychotherapy... I always see [MH apps] as a kind of substitute for situations when therapy or another person simply isn’t available.*

[P15_42F]

Although this interviewee dealt with her mental difficulties largely on her own, she perceived her difficulties as too severe to be addressed through MH apps alone. This perceived limitation was echoed by another participant, who explicitly stated:

*When... or once it becomes long-term, an app on its own won’t really help.*

[P23_24F]

Taken together, these accounts suggest that persistent reliance on MH apps may reflect not only individual coping strategies, but also structural constraints that shape access to professional MH care.

#### Not Using MH Apps When I Feel Fine: Episodic and Conditional Use

Episodic and conditional use was most commonly associated with the ill-being pattern and was characterized by nonsystematic engagement with MH apps. Participants frequently reported that they stopped using the app once acute distress subsided. Unlike trajectories toward independence, episodic and conditional use did not involve internalizing app-based practices. Instead, participants described keeping MH apps available as resources to be reactivated during periods of psychological difficulty, while preferring other self-maintenance strategies during less demanding times.

Some interviewees indicated that their MH app use followed a seasonal pattern, triggered by episodic deteriorations in well-being, typically during autumn or winter. These interviewees expressed interest in seasonal subscriptions with flexible and user-friendly terms, reflecting how their engagement with MH apps was shaped by seasonal need:

*I wouldn’t want to pay for an expensive annual subscription, especially if I know I’m not using it on a daily basis.*

[P22_23F]

This participant did not perceive daily MH app use as necessary and consequently was not interested in committing to a long-term subscription.

### Theme 3: Perceived Costs and Unintended Consequences of MH App Use

#### Overview

While most participants described MH apps as helpful (eg, providing structure, calming guidance, and distraction during stress), they also reflected on tensions and unintended consequences arising from their use. We identified 4 interrelated subthemes that captured these perceived costs. First, “*It helps, but only if I make it work*” describes tensions that stem from the perceived responsibility for making app use effective. Second, “*A tool I lean on... but when is it too much?*” captures tensions around reliance on apps, including their use as substitutes for other forms of support and the potential delay of help-seeking. Third, “*Inward focus as a risk*” highlights how self-monitoring and reflective practices, while often stabilizing, can also intensify attention to internal states and become ruminative. Finally, “*Digital paradoxes of self-care*” describes conflicts between app-based self-care and attempts to reduce screen time or disengage from stressful technologies. Across these subthemes, tensions emerged not solely because of functional limitations, but through participants’ active efforts to engage with digital self-care and their ongoing evaluations of the costs and benefits of app use.

#### It Helps, but Only if I Make It Work

Across interviews, participants repeatedly emphasized that, while MH apps offered accessible support, they could not generate the motivation required for consistent or effective use. Apps were commonly framed as passive tools whose benefits depended on users’ own effort and willingness to engage:

*[...] the apps will only remind you that this is important, but they won’t do the work for you.*

[P04_24F]

Similarly, another participant stressed that change stemmed from her own determination rather than the app itself, which merely provided a starting point:

*It’s not that the app directly forced me to take better care of my mental health. What actually pushed me to do something about it was mainly that [my mental states] were just burdensome.*

[P21_24F]

Across accounts, participants recognized a certain level of agency as a prerequisite for benefit. For some, this responsibility was framed as a natural or even empowering aspect of app use; for others, it was experienced as a burden or source of tension.

This ambivalence was particularly visible in relation to reminders and streak-based features. While some participants appreciated the structure these features provided, others described actively protecting their autonomy by resisting notifications or streak mechanisms that induced guilt or created tension between disengaging from the app and losing accumulated rewards. As 1 participant explained, reminders were acceptable only as long as they did not make her feel bad for noncompliance:

*If it made me feel bad for not doing something, I’d probably be done with this shit.*

[P04_25F]

In such cases, perceived pressure was not motivational but instead led to disengagement.

Beyond motivation and effort, some participants emphasized that benefiting from MH apps also presupposed a certain level of competence (eg, understanding and appropriately applying the techniques offered). One participant noted that merely having access to coping strategies was insufficient; through therapy, she learned how to work with techniques she had previously encountered in the app:

*[...] to get the right effect, you need to have the right techniques and know how to use them... thanks to therapy, I learned how to do that.*

[P07_23F]

Again, this reinforced the idea that healthcare providers could play a crucial role in guiding some users to successfully leverage the apps’ functions. Taken together, these findings indicate that the apps’ effectiveness is not intrinsic, but negotiated through the users’ engagement with them.

#### A Tool I Lean on… but When Is It Too Much?

Participants reported several unintended consequences related to growing reliance on MH apps, often framed as a tension between the benefits of immediate support and concerns about depending too heavily on the app. Apps were often described as psychological crutches that were helpful in moments of distress, yet accompanied by reflections on whether such reliance might limit the development or use of alternative coping strategies. One participant recalled how her partner explicitly raised this concern while observing her use of the app during anxiety-provoking situations on public transport, prompting her to consider what would happen if the app were unavailable:

*...he came up with this interesting idea... what would I do if I didn’t have my cell phone... that I actually rely on it a little bit... and so I become a little dependent on it.*

[P21_23F]

Reliance on apps was also linked to reflections on help-seeking beyond the app. Participants frequently emphasized that they themselves were responsible for recognizing when app-based support was no longer sufficient and when professional help was needed. One interviewee articulated particularly clearly how short-term relief provided by apps could intersect with postponing professional help. She first framed this as a general risk:

*...people often, instead of paying for care, reach for an app and think that it will replace therapeutic care.*

[P07_23F]

The interviewer then asked:

***Interviewer:** Is that also your experience?*

***P07_23F:** Absolutely, yes. I was also that person who reached for the app first [...] but I didn’t have any guarantee that it’s actually helping me.*

In these accounts, the perceived risk concerned the way reliance on short-term app support could reshape judgments about when additional help was necessary.

#### When Looking Inward Becomes a Problem

Some participants worried that app use promoted an inward focus, which they found emotionally demanding rather than uniformly supportive, particularly when it involved confronting negative emotions or unresolved difficulties. As one participant described, reflective prompts were not limited to positive experiences but also required deeper engagement:

*[...] that not only positive questions, but also deeper ones, where you really have to think about it and it hurts. It can hurt.*

[P02_20M]

In a similar vein, others questioned whether repeated attention to negative emotional states might reinforce rather than alleviate distress:

*If I kept feeling like shit and kept saying that I felt sick, I think I would have just continued to feel sick, because I would have kept repeating to myself that I feel sick.*

[P04_24F]

In these accounts, self-monitoring was experienced as effortful and potentially counterproductive when not accompanied by sufficient support or resolution.

Others expressed concern that sustained inward engagement could lead to withdrawal from social interaction, noting that excessive self-focus might not be desirable:

*...if I kept using it, I’d really withdraw into myself, which probably wouldn’t be good.*

[P12_34F]

Taken together, these accounts point to the double-edged nature of MH apps; while privacy and inward focus are central to digital self-care, they may also become emotionally demanding or socially isolating under certain conditions.

#### Digital Paradoxes of Self-Care

Finally, some participants reflected on broader digital paradoxes inherent in app-based self-care, highlighting tensions between the goals of mental well-being and the means through which support was delivered. Several respondents described a contradiction between using MH apps and their simultaneous efforts to reduce overall technology or screen use, particularly when digital environments themselves were experienced as stressful or overstimulating. As 1 participant noted:

*I am trying to distance myself a bit from technology right now because I’m finding it’s one of the triggers that stresses me out. Actually, meditating and using an app are perhaps a bit... maybe they go against each other a little. It just shouldn’t feel forced, you know? Which technology often does.*

[P30_43M]

Others similarly described discomfort with the necessity of keeping the phone close to access support, particularly when opening an app risked drawing them into other, unwanted digital activities:

*...it bothers me that it forced me... that I have to keep it on me... once it’s open, I start dealing with... other things there.*

[P25_42F]

In response, some participants reported avoiding app use in certain contexts, such as at work or in overstimulating environments, acknowledging that the device itself could introduce distraction or exacerbate stress.

## Discussion

### Principal Findings

In our study, we explored why users adopt MH apps and how they integrate these tools into their lives and into broader ecosystems of MH care and support. Our findings highlight that user engagement is a continual negotiation between app functionalities and users’ evolving needs. Importantly, we provide further evidence that disengagement can be a meaningful outcome of MH app use, challenging engagement-centric assumptions that dominate current app design and evaluation.

Our results suggest that sustained MH app use is perceived as a goal only in a limited set of contexts. In other situations, MH apps are understood either as tools for temporary solutions or as instruments intended to foster the development of autonomous self-care practices that eventually become independent of MH apps. Our findings show that for some users, overcoming a difficult period or successfully integrating self-care practices into daily self-care maintenance resulted in what has been previously termed “positive disengagement” [27]. As users’ goals were reached, they naturally decreased their app usage, showing that disengagement may follow individuals’ own appraisals that the app is no longer needed. These reported experiences challenge the existing body of research on MH apps and the problem of low user engagement [9,14,15] by demonstrating that for some users, long-term sustained engagement is in direct conflict with their goals.

Beyond goal attainment, disengagement also emerged as a protective strategy in what we describe as the *digital self-care paradox*, whereby users intentionally reduce their engagement with digital tools to minimize the negative effects associated with prolonged technology use, such as overreliance on MH apps [19,23] or technology fatigue [15]. Moreover, usage discontinuation was often the result of successful substitution for off-screen self-care behaviors (eg, spending time outside). Therefore, “episodic” use or abandonment can often be a sign of a procedural recalibration of resources toward nondigital self-care, indicating successful self-care management.

This study showed that users varied considerably in their initial motivational patterns, which coincided with different self-care phases described in the MRTSC [26]. These patterns evolved along the following two dimensions: (1) intent, which referred to whether MH app use was oriented towards supporting well-being (emphasizing self-care maintenance), addressing ill-being (driven by self-care management), or understanding a specific problem (through self-care monitoring); and (2) engagement timing, which reflected when and how the apps were used, ranging from habitual and episodic to reactive use. Additionally, our findings showed that these initial patterns evolved over time into distinct trajectories shaped by the users’ needs and resources, including enhanced self-care management supported by MH apps (self-emancipatory trajectories), the development of autonomous self-care without continued reliance on MH apps, increased reliance on MH apps as substitutes for human support, and episodic use limited to periods of perceived need.

Our findings provide further evidence of how MH apps enhance self-care maintenance, particularly through well-being–oriented and habitual use aimed at promoting psychological stability. Specifically, MH apps contribute to self-maintenance by legitimizing space for self-care, maintaining self-care routines, or supporting activities that reduce overall stress. The MRTSC suggests that the value of these digital routines lies in their cognitive economics. Ideally, apps offload the cognitive burden of daily maintenance, preserving users’ psychological resources. However, our data reveal a critical tension—the digital mechanisms designed to support these habits (eg, notifications or streaks) may backfire. Instead of seamlessly integrating self-care into daily life, these intrusive features paradoxically increased technology fatigue, disrupting the very processes they aimed to facilitate.

Self-monitoring constituted a central component of the problem-oriented pattern of MH app use, although it was also present within the well-being–oriented and habitual patterns. While the MRTSC conceptualizes self-care monitoring as the observation and interpretation of changes, our findings illustrate that in the context of digital MH, this process extends beyond discrete symptom tracking. Some users engaged in detailed journaling and broader self-narrativizing. Here, the app facilitated a highly complex form of appraisal, allowing users to contextualize their psychological states or alternatively record positive experiences to counterbalance negative affect and keep a “life history.” Crucially, however, the effectiveness and perception of this meaning-making depended on intentionality. When tied to a specific, personally meaningful goal, monitoring was beneficial. Conversely, when self-monitoring lacked clear intent, it was frequently experienced as burdensome or even depressing, which is consistent with prior research on the adverse outcomes of MH app use [10]. Ultimately, our data expand on this finding, suggesting that the value of digital self-monitoring hinges less on the mechanical act of tracking and more on the user’s finding meaning in the process of recording data (ie, on their capacity to articulate why and how monitoring is meaningful for them).

Our study engages with the implications of stand-alone digital technologies designed for private and autonomous use by showing that MH apps were indeed largely managed by users themselves and rarely embedded in shared or professionally supported care practices. One strand of our findings illustrates how MH apps facilitated the processes of self-emancipation, supporting transitions from professionally delivered treatment to self-care. At the same time, our results show that external feedback on MH app use, whether from close others or health care professionals, often enhanced engagement, enabling users to refine their usage patterns or to apply skills acquired through the apps beyond the screen. Such external support proved especially vital for users who reported that in times of acute psychological distress, it was difficult to apply the techniques presented within the app. This finding resonates with the work of Villegas Mejía et al [25], who emphasized the importance of supporting engagement that extends beyond the MH app context.

Consistent with prior research [22], our interviews revealed numerous examples of MH app use enhancing self-care management by fostering skills and self-care habits that enabled some participants to transition from professionally delivered treatment to more autonomous management of their MH. However, this self-enhancement may have unintended consequences. For some users, greater perceived autonomy in self-management was accompanied by a heightened sense of personal responsibility for managing MH difficulties, leading them to hesitate to seek professional help, out of concern that they might burden health care providers or divert resources from those in greater need. Viewed through the MRTSC, this hesitation is notable, as the theory positions consultation and seeking information as adaptive self-care management responses rather than departures from self-care. In treating help-seeking as a failure of autonomy, these users adopted a framing at odds with the MRTSC, in which turning to others is itself a legitimate self-care behavior. Our findings also resonate with the research of Van der Poll et al [24], who similarly described how MH app users may willingly assume personal responsibility for managing their own MH. Extending their work, our data suggest that this sense of responsibility can be accompanied by increased hesitation to seek professional help.

Moreover, our findings suggest that some users develop reliance on MH app–related practices as a primary source of immediate relief from negative emotional states. Although rapid relief may prevent symptoms from escalating, it may reduce the likelihood that users reflect on the seriousness of their condition and consider seeking professional help, potentially sustaining reactive crisis management. Unlike crisis hotlines, most stand-alone MH apps do not include mechanisms that actively encourage or facilitate seeking professional support.

We consider these findings especially concerning in populations where barriers to MH care are already high. In such contexts, promoting MH apps as a solution risks obscuring the problem in the short term while exacerbating it in the long term. Individuals who might otherwise seek professional help may instead rely on an app as a form of temporary relief, making symptoms more manageable while foregoing appropriate treatment. While this relief may reduce immediate distress, it can also prolong avoidable suffering and leave underlying needs for care unmet. What may appear to be a solution to limited access to MH services may, in fact, function only as a palliative response to a broader structural problem.

### Limitations 

This study has several limitations. First, the distinction between “well-being” and “ill-being” orientations may partly reflect processes of self-narrativization, whereby participants construct coherent accounts of their MH app use that align with identity work rather than their immediate psychological states. Second, the study relies on retrospective accounts of MH app use. Participants reconstructed the evolution of their engagement over time based on memory and subjective interpretation, which may introduce recall bias. Future research would benefit from longitudinal designs capable of capturing trajectories of MH app use as they unfold. Third, although our analysis focused on MH apps, some participants reported extending their digital self-care practices by consulting generative AI tools (eg, large language models) embedded in, or external to MH apps.

Finally, the sample was relatively homogeneous, consisting predominantly of young, female, Czech, digitally engaged participants, which limits the range of experiences represented and, consequently, the transferability of the findings to other populations and contexts. In addition, individuals currently experiencing a psychological crisis were excluded from participation. Our findings may therefore not fully capture experiences of MH app use during acute psychological distress. Future research should examine whether and how the identified patterns and trajectories manifest across more diverse demographic, cultural, and clinical contexts.

### Recommendations

Similar to health care providers, MH app designers may consider “positive disengagement” as a legitimate outcome. Because some app designs discourage this through various mechanisms (eg, daily streaks), incorporating “pause” functions, allowing flexible payment plans, and avoiding punitive gamification mechanics may empower users to successfully integrate coping strategies off-app. MH apps could also be designed to promote connection rather than substitute for human support. Instead of functioning as self-contained solutions, apps might serve as intermediary or boundary objects that facilitate communication with professionals, counselors, or trusted peers. Moreover, apps with emotion tracking or crisis support features may integrate empathic prompts to direct users to low-barrier resources (eg, hotlines and MH professional directories) in response to the detection of sustained distress. Clear guidance to distinguishing between manageable distress and symptoms that warrant clinical attention, alongside normalizing help-seeking as a legitimate and responsible step, may reduce feelings of shame and prevent responsibilization for solving one’s own problems. In this way, MH apps can function as bridges to care rather than barriers to professional support.

For clinicians, the practical task is to bring clients’ motivations for autonomous app use into the conversation and help educate them about the limits of these tools. Clients prone to reactive use during acute distress warrant particular attention, as these apps rarely offer crisis plans. MH professionals should work with such clients on a crisis plan that does not depend on the app. Mood tracking and journaling should be encouraged only for clients who experience them as beneficial or connected to personally meaningful goals.

### Conclusion

This study shows how MH apps are embedded within broader practices of care and support, and under which conditions they may be empowering or problematic. For some users, they foster autonomy, strengthen self-care skills, and enable the transfer of coping strategies beyond digital platforms and even beyond professional treatment. In such cases, discontinuing app use may reflect the successful integration of self-care into everyday life. For others, however, MH apps may reinforce app-based coping and contribute to reliance on digital tools. Overall, providing clearer distinctions between everyday emotional strain and clinically significant symptoms, while framing help-seeking as a responsible and legitimate course of action, may help alleviate shame and counteract the tendency to place excessive responsibility on individuals to manage their own MH.

## Supplementary material

10.2196/95414 Multimedia Appendix 1 English translation of the interview questions.

10.2196/95414 Multimedia Appendix 2 Positionality.

10.2196/95414 Checklist 1 COREQ checklist.

## Abbreviations

ADHD: attention-deficit/hyperactivity disorder
COREQ: Consolidated Criteria for Reporting Qualitative Research
MH: mental health
MRTSC: Middle-Range Theory of Self-Care of Chronic Illness
RQ: research question
RTA: reflexive thematic analysis
