# Supplementary material for: How People Integrate Mental Health Apps Into Everyday Self-Care: Qualitative Study
Source: JMIR Mhealth Uhealth. 2026 Oct 9;14:e95414. doi: 10.2196/95414 (PMC13652382; doi:10.2196/95414)
Supplement: Supplementary file 1 [file mhealth-v14-e95414-s002.pdf]

Positionality

VS engaged with this study as a junior public health psychology researcher with a critical orientation. He approached the subject with personal experience of mindfulness applications that teach practice through guided sessions, though his own practice has since shifted toward a basic timer application without guidance. He believes that his position is that of a partial outsider as his mindfulness practice represents only a narrow segment of the broader population of mental health application users. His experience of these tools has been largely positive, and he recognizes their potential to lower barriers to psychoeducation and self-care. At the same time, his critical background informs a reserved stance toward the commercialization of wellbeing. He is attentive to the absence of professional oversight, and to the ways commercial incentives can lead developers to treat marketing as more consequential than the product itself.

ZS approached the study from a critical psychology perspective and contributed a critical realist orientation and extensive experience in qualitative research. Her perspective sensitized her to the individualization of responsibility for mental health and well-being, including cultural expectations that individuals should independently manage distress, remain productive, and treat negative emotional states as problems to be overcome. She was particularly attentive to the tension between the potential value of self-management tools and the possibility that they may shift responsibility for care toward individuals and away from interpersonal/community or professional sources of support. She approached participants' experiences with genuine curiosity. ZS had limited and primarily instrumental experience with mental health apps, having used a meditation application to support sleep and an exercise application to facilitate routine. She viewed these as supplementary tools rather than central components of self-care. Her broader understanding of mental health was informed by personal familiarity with therapy, journaling, and other forms of self-care and by a positive orientation toward seeking professional support. She initially anticipated relatively straightforward accounts of when and why MH apps were helpful or problematic. Participants' accounts challenged this expectation, revealing more nuanced and varied meanings of autonomy, responsibility, engagement, and disengagement.

AŠ approached this research as a critical realist with extensive experience in qualitative research on health behavior. Having no direct personal experience with mental health apps, she approached the topic with curiosity about how these digital technologies are incorporated into everyday self-care practices and situated within broader health care systems. She was particularly interested in the boundaries between self-care and professional care, including how responsibility for mental health is negotiated across these domains. As the principal investigator of the project On Our Own: Opportunities and Risks in the Individualization of Society (PRINS), AŠ was sensitized to questions concerning both the opportunities and potential risks associated with increasing individual responsibility for health and well-being. This perspective led her to attend to both positive experiences of MH app use and potential unintended consequences, including the ways in which such technologies may reshape expectations regarding self-care and professional support. Rather than assuming that MH apps are either beneficial or problematic, she approached participants' accounts with an interest in understanding how these tools are embedded in broader systems of care and how users make sense of their role in managing mental health.
