# Supplementary material for: How People Integrate Mental Health Apps Into Everyday Self-Care: Qualitative Study
Source: JMIR Mhealth Uhealth. 2026 Oct 9;14:e95414. doi: 10.2196/95414 (PMC13652382; doi:10.2196/95414)
Supplement: Supplementary file 2 [file mhealth-v14-e95414-s003.pdf]

No	Item	Guide questions/description	
**Domain 1: Research team and reflexivity**			
Personal Characteristics			
1.	Interviewer/facilitator	Which author/s conducted the interview or focus group?	All article authors
2.	Credentials	What were the researcher's credentials? *E.g. PhD, MD*	doc., Ph.D., Mgr., 3x Bc., all in psychology
3.	Occupation	What was their occupation at the time of the study?	Professor, psychology researchers, students
4.	Gender	Was the researcher male or female?	4x Female, 2x Male
5.	Experience and training	What experience or training did the researcher have?	Senior researchers had extensive experience and training in qualitative research; student interviewers received training in qualitative interviewing.
Relationship with participants			
6.	Relationship established	Was a relationship established prior to study commencement?	No
7.	Participant knowledge of the interviewer	What did the participants know about the researcher? e.*g. personal goals, reasons for doing the research*	See Method
8.	Interviewer characteristics	What characteristics were reported about the interviewer/facilitator? e.g. *Bias, assumptions, reasons and interests in the research topic*	See Method – positionality, Suplement3
**Domain 2: study design**			
Theoretical framework			
9.	Methodological orientation and Theory	What methodological orientation was stated to underpin the study? *e.g. grounded theory, discourse analysis, ethnography, phenomenology, content analysis*	Reflexive thematic analysis
Participant selection			

No	Item	Guide questions/description	
10.	Sampling	How were participants selected? *e.g. purposive*, *convenience, consecutive, snowball*	convenience, snowball
11.	Method of approach	How were participants approached? e*.g. face-to-face, telephone, mail, email*	Social media ads, face-to-face, email
12.	Sample size	How many participants were in the study?	31
13.	Non-participation	How many people refused to participate or dropped out? Reasons?	None
Setting			
14.	Setting of data collection	Where was the data collected? e*.g. home, clinic, workplace*	Research institute or at home. Always in a private setting.
15.	Presence of non-participants	Was anyone else present besides the participants and researchers?	No
16.	Description of sample	What are the important characteristics of the sample? *e.g. demographic data, date*	See Table 1
Data collection			
17.	Interview guide	Were questions, prompts, guides provided by the authors? Was it pilot tested?	Yes, see Appendix. Yes, each interviewer piloted the questions on at least 1 person
18.	Repeat interviews	Were repeat interviews carried out? If yes, how many?	No
19.	Audio/visual recording	Did the research use audio or visual recording to collect the data?	Audio recording
20.	Field notes	Were field notes made during and/or after the interview or focus group?	Yes
21.	Duration	What was the duration of the interviews or focus group?	See Table 1.
22.	Data saturation	Was data saturation discussed?	No
23.	Transcripts returned	Were transcripts returned to participants for comment and/or correction?	No
**Domain 3: analysis and findings**z			
Data analysis			
24.	Number of data coders	How many data coders coded the data?	3
25.	Description of the coding tree	Did authors provide a description of the coding tree?	No
26.	Derivation of themes	Were themes identified in advance or derived from the data?	Derived
27.	Software	What software, if applicable, was used to manage the data?	MaxQDA

No	Item	Guide questions/description	
28.	Participant checking	Did participants provide feedback on the findings?	No
Reporting			
29.	Quotations presented	Were participant quotations presented to illustrate the themes / findings? Was each quotation identified? e.*g. participant number*	Yes. Yes.
30.	Data and findings consistent	Was there consistency between the data presented and the findings?	Yes
31.	Clarity of major themes	Were major themes clearly presented in the findings?	Yes
32.	Clarity of minor themes	Is there a description of diverse cases or discussion of minor themes?	Yes
