# Supplementary material for: How People Integrate Mental Health Apps Into Everyday Self-Care: Qualitative Study
Source: JMIR Mhealth Uhealth. 2026 Oct 9;14:e95414. doi: 10.2196/95414 (PMC13652382; doi:10.2196/95414)
Supplement: Supplementary file 3 [file mhealth-v14-e95414-s001.docx]

**English Translation of the Interview Questions**

**1. Introduction and Context of Use**

- How did you hear about this app and what led you to download it?
- How long and how often do you use it now?
- Under what circumstances or in what situations do you use the app?
- Do you talk to anyone around you about using the app? How do they react?

**2. First Impressions and Expectations**

- What was your first impression of the app?
- What expectations did you have from the app?

**3. Motivations and Needs**

- In what situations did you think the app could be useful to you?
- What motivates or demotivates you to continue using it?

**4. Comparison with Other Mental Health Care Methods**

- What is your experience with other methods of mental health support?
  - *E.g., private therapy, journaling, meditation, breathing exercises, self-help books, podcasts, support groups...*
- In what ways is the app better or worse than other mental health care options specifically for you?
- Is there any type of support that you would have previously sought elsewhere, and which the app now fully substitutes?

**5. Benefits, Usefulness, Positive Experience**

- In what specific situations did you feel the app helped you the most?
- Do you remember a moment when the app pleasantly surprised you? What was that experience like?
- What exactly do you value about its content or features?

**6. Negative Experience, Doubts, Critical Moments**

- Have you ever experienced a situation where the app disappointed or irritated you? What happened?
- Have you ever doubted the expertise or effectiveness of the app?
  - How did you handle those doubts?
- Can you recall a moment when the app did not work well for your current needs? How did you react in that moment?

**7. Personal Significance of the App**

- What emotions does using the app usually evoke in you?
- What role would you say the app plays in your life?
- If the app were to disappear, what would you miss the most?
- What words or metaphors would you use to describe the app's significance to you?

**8. Long-term Horizon: Perception of Care, Habits, Experiences**

- How has your attitude towards mental hygiene habits changed over time?
- Were there periods when you stopped using the app or reduced your frequency of use? Why?
- What impact has using the app had on your motivation to care for your mental health?
- Has your attitude towards other forms of mental health care changed in any way since you started using or used the app?

**9. Future, Recommendations**

- What changes or new features would you welcome in the app in the future?
- For what kind of people do you think the app is suitable, and for whom is it unsuitable?

**10. Trust in Developers and Data Handling**

- What kind of relationship do you think the app's developers have with its users?
  - What makes you think that?
- How do you think the app handles your data?
  - What data provided to the app do you consider personal?
- What should or shouldn't the developers do to make their app as trustworthy as possible for you?

**11. Closing Remarks**

- Is there anything else you think we haven't covered in our interview that you'd like to share?
- Do you have any advice or feedback for me as a researcher regarding the format or content of our interview?

**Original Czech Interview Questions**

1. **Úvod a kontext používání**

- Jak jste se o této aplikaci dozvěděl/a a co vás vedlo k jejímu stažení?
- Jak dlouho a jak často ji nyní používáte?
- Za jakých okolností nebo v jakých situacích aplikaci používáte?
- Mluvíte s někým ze svého okolí o tom, že používáte aplikaci? Jak na to reagují?

2. **První dojmy a očekávání**

- Jaký byl váš první dojem z aplikace?
- Jaká očekávání jste od aplikace měl/a?

3. **Motivace a potřeby**

- V jakých situacích jste si myslel/a, že by vám aplikace mohla být užitečná?
- Co vás motivuje nebo demotivuje pokračovat v jejím používání?

4. **Srovnání s jinými způsoby péče o duševní zdraví**

- Jaké máte zkušenosti s jinými způsoby podpory duševního zdraví?
  - *Např. soukromá terapie, deníkování, meditace, dechová cvičení, svépomocné knihy, podcasty, skupiny...*
- V čem je aplikace lepší nebo horší než ostatní možnosti péče o duševní zdraví právě pro vás?
- Existuje nějaký druh podpory, který byste dříve hledal/a jinde, a nyní to plně supluje aplikace?

5. **Přínosy, užitečnost, pozitivní zkušenost**

- V jakých konkrétních situacích jste pocítil/a, že vám aplikace nejvíc pomohla?
- Vzpomínáte si na moment, kdy vás aplikace příjemně překvapila? Jaký to byl zážitek?
- Čeho konkrétně si ceníte na jejím obsahu nebo jejích funkcích?

6. **Negativní zkušenost, pochybnosti, kritické momenty**

- Zažil/a jste někdy situaci, kdy vás aplikace zklamala nebo iritovala? Co se stalo?
- Pochyboval/a jste někdy o odbornosti či efektivitě aplikace?
  - Jak jste s pochybnostmi nakládal/a?
- Vzpomenete si na okamžik, kdy aplikace nefungovala dobře pro vaše aktuální potřeby? Jak jste v tu chvíli reagoval/a?

7. **Osobní význam aplikace**

- Jaké emoce ve vás používání aplikace obvykle vyvolává?
- Jakou roli byste řekl/a, že má aplikace ve vašem životě?
- Kdyby aplikace zmizela, co byste nejvíce postrádal/a?
- Jakými slovy nebo metaforami byste popsali význam aplikace pro vás?

8. **Dlouhodobý horizont: vnímání péče, návyky, zkušenosti**

- Jak se váš postoj k návykům spojeným s duševní hygienou měnil v průběhu času?
- Byla období, kdy jste aplikaci přestal/a používat nebo omezil/a její frekvenci? Proč?
- Jaký vliv mělo používání aplikace na vaši motivaci pečovat o duševní zdraví?
- Změnil se nějak váš postoj k ostatním formám péče o duševní zdraví od doby, co aplikaci používáte nebo jste používal/a?

9. **Budoucnost, doporučení**

- Jaké změny či nové funkce byste v aplikaci uvítal/a do budoucna?
- Pro jaké lidi si myslíte, že je aplikace vhodná a pro jaké zas naopak nevhodná?

10. **Důvěra ve vývojáře a nakládání s daty**

- Jaký vztah si myslíte, že mají vývojáři aplikace k jejím uživatelům?
  - Na základě čeho si to myslíte?
- Jak si myslíte, že aplikace nakládá s vašimi daty?
  - Jaká data poskytovaná aplikaci považujete za osobní?
- Co by vývojáři měli nebo naopak neměli dělat, aby pro vás byla jejich aplikace co nejvíce důvěryhodná?

11. **Volný závěr**

- Je ještě něco, co podle vás v našem rozhovoru nezaznělo, a o co byste se chtěl/a podělit?
- Máte pro mě jako výzkumnici/níka nějakou radu nebo zpětnou vazbu k formátu nebo obsahu našeho rozhovoru?
